# Supplementary material for: Quantitative prediction of disinfectant tolerance in Listeria monocytogenes using whole genome sequencing and machine learning
Source: Sci Rep. 2025 Mar 26;15:10382. doi: 10.1038/s41598-025-94321-6 (PMC11947258; doi:10.1038/s41598-025-94321-6)
Supplement: Supplementary file 2 — Supplementary Material 2 [file 41598_2025_94321_MOESM2_ESM.docx]

***S1 Appendix:*** *Description of SNPs feature extraction workflow.*


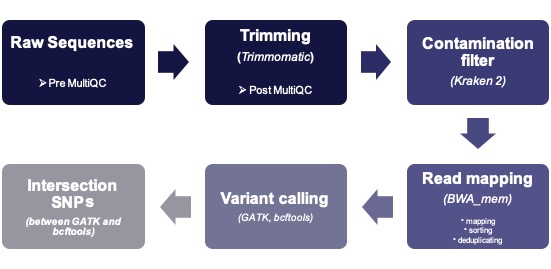


*Figure 1: Workflow diagram*

For initial assessment of the raw reads we used FastQC (v0.11.9) [1] and MultiQC (v1.12) [2]. Adapter clipping and quality trimming was performed using Trimmomatic (v0.38) [3] specifying ‘ILLUMINACLIP:adapters.fa:4:30:10:2:keepBothReads’ and ‘LEADING:3 TRAILING:3 SLIDINGWINDOW:4:20 MINLEN:50’ respectively. The post trimming quality of the raw reads was evaluated the same way as for the initial assessment. In order to identify contaminations, the trimmed reads were assigned taxonomic labels with Kraken (v2.1.2) [4]. The reads where then filtered according to NCBI Taxonomy ID along the taxonomic tree until the *L. monocytogenes* species level (i.e., Bacteria (NCBI:txid2); Terrabacteria group (NCBI:txid1783272); Firmicutes (NCBI:txid1239); Bacilli (NCBI:txid91061); Bacillales (NCBI:txid1385); Listeriaceae (NCBI:txid186820); Listeria (NCBI:txid1637); L. monocytogenes (NCBI:txid1639)). After contamination filtering we mapped the reads to the EGD-e reference genome (Accession: GCF_000196035.1) using BWA-MEM (v0.7.10) [5]. The mapping results were quality checked and improperly paired reads as well as paired reads that have a mapQ score below 30 were excluded using SAMtools (v1.14) [6]. The mapped reads were then sorted and duplicates were marked with Picard (v2.26.10) [7]. Finally, we used two different tools GATK (v4.2.6.1) [8] and BCFtools (v1.14) [6] to call single nucleotide variants (SNVs). To get a more reliable variant calling result we are combining the output from both variant callers and find the intersection of both outputs, i.e., we are only keeping SNVs that are called by both tools. The results from the SNV calling workflow are subsequently used as input for the machine learning.

***S2 Appendix:*** *Description of pan-genome gene cluster feature extraction workflow.*

In short, raw reads are trimmed with bbduk2 from BBTools (v36.49) [9] using only reads with length ≥50 bp and Phred score per base ≥20 (from right to left), and filtering the institution-specific adapters. Trimmed reads were quality checked using FastQC (v0.11.5) [1] and assembled using SPAdes (v3.11.0) [10] with a k-mer coverage of two and excluding contigs <500 bp. All isolate’ sequence assemblies were annotated using Prokka (v1.14.5) [11] specifying the ‘--kingdom Bacteria’ and ‘--genus Listeria’ flag. The annotated sequences where then used to build a pan-genome with Roary (v3.13.0) [12] using the flags for multiFASTA alignment of core genes using PRANK (v140603) (‘-e’) [13] and fast core gene alignment with MAFFT (v6.864b) (‘-n’) [14]. All assemblies were then screened for presence of the pan-genome gene clusters obtained from Roary (i.e., each gene cluster has a representative chosen by Roary) using tblastx from ncbi-blast suit (v2.13.0+) [15] with pan-genome reference as ‘-query’, the assemblies as ‘-subject’, setting ‘-evalue’ to 0.001, and ‘-max_hsps’ to 1.

***S3 Appendix:*** *List of ML models used for the pre-screening and the respective scikit-learn class instance used.*

**Classification:**

Logistic Regression L1 (sklearn.linear_model.LogisticRegression)

Logistic Regression L2 (sklearn.linear_model.LogisticRegression)

Logistic Regression ElasticNet (sklearn.linear_model.LogisticRegression)

Stochastic Gradient Descent L1 (sklearn.linear_model.SGDClassifier)

Stochastic Gradient Descent L2 (sklearn.linear_model.SGDClassifier)

Stochastic Gradient Descent ElasticNet (sklearn.linear_model.SGDClassifier)

Decision Tree (sklearn.tree.DecisionTreeClassifier)

Random Forest (sklearn.ensemble.RandomForestClassifier)

Extra Trees (sklearn.ensemble.ExtraTreesClassifier)

AdaBoost Trees (sklearn.ensemble.AdaBoostClassifier)

Gradient Boosting Trees (sklearn.ensemble.GradientBoostingClassifier)

Complement NB (sklearn.naive_bayes.ComplementNB)

Support Vector Machine L1 (sklearn.svm.LinearSVC)

Support Vector Machine L2 (sklearn.svm.LinearSVC)

Dummy Classifier (sklearn.dummy.DummyClassifier)

**Regression:**

Linear Regression (sklearn.linear_model.LinearRegression)

Linear Regression L1 (sklearn.linear_model.Lasso)

Linear Regression L2 (sklearn.linear_model.Ridge)

Linear Regression ElasticNet (sklearn.linear_model.ElasticNet)

Support Vector Machine RBF (sklearn.svm.SVR)

Gradient Boosting Trees (sklearn.ensemble.GradientBoostingRegressor)

Random Forest (sklearn.ensemble.RandomForestRegressor)

Dummy Regressor (sklearn.dummy.DummyRegressor)

***S4 Appendix:*** *Description of feature extraction from the validation set data.*

To prepare the genome sequences for prediction with our pre-trained model, we extracted the required features by aligning the pan-genome gene cluster references against the sequences. The validation set consists out of both, raw read and assembly data. To avoid computationally expensive de-novo assembly, we are using KMA (v1.3.15) [16] to align the pan-genome genes to the raw reads. For the already assembled part of our validation set, we are using tblastx (v2.13.0) [15] to align the reference database.

***S5 Appendix:*** *Manual annotation for the important features.*

To gain further knowledge about possible functions of the important features, we screened the pan-genome gene clusters against a non-redundant database of known annotations. To make this database, we collected coding sequence (CDS) annotations from complete reference genomes (n=318) in NCBI’s RefSeq database. CDS duplicates were filtered using SeqKit (v2.5.1) [17]. The sequences of the ten most important features where then aligned against the annotation database using tblastx (v2.13.0+) [15] and the best matches were reported in Table S5. A list with included RefSeq accession numbers can be found on (<https://github.com/agmei/LmonoDisinfectML>).

***S6 Appendix:*** *further investigation of low performance for Palma et al. test set .*

To better understand for which isolates our models fails to make correct predictions, we compared the “actual” phenotype classes (i.e. tolerant/ susceptible classes according to a MIC threshold), and predicted phenotype classes for the Palma et al. dataset (Table S2). We found that most of the misclassified isolates corresponded to MIC values of 1,25 mg/L which is the threshold to distinguish between tolerant and susceptible classes. Mapping the “actual” and predicted phenotype classes against the screening results of the known QAC resistance genes, we could see that only a few resistant isolates (11 out of 105) harboured one of the QAC genes. We could also see that the ML-predicted phenotypes are almost in perfect concordance with the absence/presence of QAC genes.

1. Andrews S. FastQC:  A quality control tool for high throughput sequence data. 2010 [cited 10 Feb 2023]. Available: <http://www.bioinformatics.babraham.ac.uk/projects/fastqc/>

2. Ewels P, Magnusson M, Lundin S, Käller M. MultiQC: summarize analysis results for multiple tools and samples in a single report. Bioinformatics. 2016;32: 3047–3048. doi:10.1093/bioinformatics/btw354

3. Bolger AM, Lohse M, Usadel B. Trimmomatic: a flexible trimmer for Illumina sequence data. Bioinformatics. 2014;30: 2114–2120. doi:10.1093/bioinformatics/btu170

4. Wood DE, Lu J, Langmead B. Improved metagenomic analysis with Kraken 2. Genome Biol. 2019;20: 257. doi:10.1186/s13059-019-1891-0

5. Li H. Aligning sequence reads, clone sequences and assembly contigs with BWA-MEM. arXiv. 2013. doi:10.48550/arxiv.1303.3997

6. Danecek P, Bonfield JK, Liddle J, Marshall J, Ohan V, Pollard MO, et al. Twelve years of SAMtools and BCFtools. GigaScience. 2021;10: giab008. doi:10.1093/gigascience/giab008

7. Picard. [cited 11 Oct 2023]. Available: <http://broadinstitute.github.io/picard/>

8. McKenna A, Hanna M, Banks E, Sivachenko A, Cibulskis K, Kernytsky A, et al. The Genome Analysis Toolkit: A MapReduce framework for analyzing next-generation DNA sequencing data. Genome Res. 2010;20: 1297–1303. doi:10.1101/gr.107524.110

9. Bushnell B. BBtools. 2022 [cited 10 Feb 2023]. Available: <https://jgi.doe.gov/data-and-tools/software-tools/bbtools/>

10. Prjibelski A, Antipov D, Meleshko D, Lapidus A, Korobeynikov A. Using SPAdes De Novo Assembler. Curr Protoc Bioinform. 2020;70: e102. doi:10.1002/cpbi.102

11. Seemann T. Prokka: rapid prokaryotic genome annotation. Bioinformatics. 2014;30: 2068–2069. doi:10.1093/bioinformatics/btu153

12. Page AJ, Cummins CA, Hunt M, Wong VK, Reuter S, Holden MTG, et al. Roary: rapid large-scale prokaryote pan genome analysis. Bioinformatics. 2015;31: 3691–3693. doi:10.1093/bioinformatics/btv421

13. Löytynoja A. Phylogeny-aware alignment with PRANK. Methods Mol Biol (Clifton, NJ). 2013;1079: 155–70. doi:10.1007/978-1-62703-646-7_10

14. Katoh K, Standley DM. MAFFT Multiple Sequence Alignment Software Version 7: Improvements in Performance and Usability. Mol Biol Evol. 2013;30: 772–780. doi:10.1093/molbev/mst010

15. Camacho C, Coulouris G, Avagyan V, Ma N, Papadopoulos J, Bealer K, et al. BLAST+: architecture and applications. Bmc Bioinformatics. 2009;10: 421. doi:10.1186/1471-2105-10-421

16. Clausen PTLC, Aarestrup FM, Lund O. Rapid and precise alignment of raw reads against redundant databases with KMA. Bmc Bioinformatics. 2018;19: 307. doi:10.1186/s12859-018-2336-6

17. Shen W, Le S, Li Y, Hu F. SeqKit: A Cross-Platform and Ultrafast Toolkit for FASTA/Q File Manipulation. PLoS ONE. 2016;11: e0163962. doi:10.1371/journal.pone.0163962
